# Chemical Secretion Use Varies Among Species but Lacks a Straightforward Relationship With Other Defenses in Neotropical Opiliones

**DOI:** 10.1002/ece3.74037

**Published:** 2026-07-19

**Authors:** Marilia Freire, Noah Skelly, Sejal V. Prachand, Ignacio Escalante

**Affiliations:** ^1^ Department of Biological Sciences University of Illinois Chicago Illinois USA; ^2^ Department of Forest Management University of Montana Missoula Montana USA

**Keywords:** chemical secretions, group living, mixed‐species aggregation, Neotropical arthropods, *Prionostemma*

## Abstract

Chemical secretion deployment is a widespread defensive strategy in arthropods, typically aimed at surviving encounters with predators. However, the ecological forces shaping variation in its use across species remain unclear. We quantified chemical secretion behavior in 11 sympatric species of *Prionostemma* within the understudied arachnid order Opiliones. We surveyed species in two distinct forests in Costa Rica and tested their secretion propensity when stimulated, and whether these differences correlate with interspecific variation in morphology, leg‐loss levels, or social behavior. We found that species varied in their tendency to release chemical secretions, forming a continuous gradient from taxa that rarely secreted to those that secreted in nearly all encounters. However, this variation was not explained by body size or leg loss. Species with similar morphology or comparable leg loss displayed contrasting patterns of secretion. Among the seven sympatric species with available social data, aggregation patterns showed no straightforward association with secretion propensity: species spanned the full range of secretion behavior, regardless of whether they frequently formed roosting aggregations with conspecifics or heterospecifics or roosted alone. Instead, each species combined secretion propensity, leg loss, body size, and aggregation tendencies in distinct ways, indicating a mosaic of defensive strategies rather than a single axis of trait covariation. Together, these results suggest that chemical defense in this group of tropical arachnids is evolutionarily labile and shaped by species‐specific ecological and social histories. This work also provides a foundation for future research on the chemical and behavioral mechanisms underlying chemical defensive diversification in arthropods.

## Graphical abstract

We asked whether variation in the willingness of congeneric species to secrete chemical defenses is associated with high between‐species differences in body size, levels of leg loss (as a proxy for engagement in another defensive strategy), and participation in social roosting with conspecifics and congenerics. We found that 11 species exhibit secretion propensity that varies along a continuous gradient, which does not correspond to simple morphological, leg‐loss, or social predictors. Species spanned a full range of responses, from taxa that rarely secreted during standardized tactile stimulation to those that secreted in every trial.

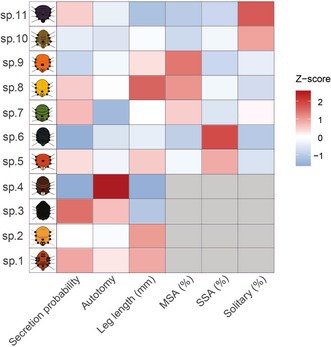

## 1 Introduction

Animals employ many strategies to reduce predation risk, including morphological defenses such as spines, armor, and aposematic coloration, behaviors such as crypsis, rapid escape, vigilance, or thanatosis, and chemical defenses in the form of externally released compounds (Edmunds 1974; Ruxton et al. 2018). Vertebrates and invertebrates alike may also rely on collective defensive behaviors such as mobbing or group formation (Edmunds 1974; Uetz et al. 2002; Caro 2005; Dittmann and Schausberger 2017). Across taxa, defensive traits and strategies often operate in combination, and species or individuals may use different ones depending on their ecological context, physiology, or life history stage (Edmunds 1974; Kikuchi et al. 2023). Chemical defenses represent one such strategy and are a widespread form of antipredator defense across animal taxa.

Among chemical defensive strategies, the use of externally released chemical secretions is particularly versatile (Eisner and Meinwald 1966; Blum 1981; Pasteels et al. 1983). Such secretions occur across numerous animal lineages and can serve multiple functions beyond predator deterrence, including competition, mate attraction, territorial marking, and social communication (Eisner and Meinwald 1966; Blum 1981; Hoffmann et al. 2006; Wertheim et al. 2005; Boulay et al. 2019). The deployment of these exocrine products can be influenced by predator pressure, physiological constraints, ecological interactions, and behavioral context. In consequence, even sympatric and closely related species may differ in their tendency to use chemical secretions and in the situations that elicit them (Chuah et al. 1989; Hoffmann et al. 2006; Krajicek et al. 2016; Boulay et al. 2019). However, the factors that drive interspecific variation in the defensive use of chemical secretions remain poorly understood, particularly how secretion propensity interacts with other defensive traits.

Several factors may contribute to variation in the use of chemical defenses. In predator deterrence, chemical secretions in arthropods can act as toxins, repellents, adhesives, or irritants (Roth and Eisner 1962; Eisner and Meinwald 1966; Dettner 1987; Pentzold et al. 2016). These secretions typically consist of complex mixtures of compounds that impair predators or reduce the likelihood of attack (Eisner and Meinwald 1966; Pasteels et al. 1983). Their use may vary across taxa or individuals and can be shaped by physiological costs associated with producing and replenishing secretions (Jones et al. 1986; Fischer et al. 2020; Boevé 2021). An individual's energetic state or competing demands may further modulate whether and when secretions are released (Zvereva and Kozlov 2016; Nazareth et al. 2016; Krajicek et al. 2016; Parvizi et al. 2018; Lindstedt et al. 2020; Fischer et al. 2020). For example, assassin bugs can adjust venom deployment based on the ecological context of an interaction, potentially conserving costly secretions when full deployment is not necessary (Fischer et al. 2020). As a result, chemical defenses rarely evolve in isolation but instead form part of broader defensive phenotypes that integrate morphological, behavioral, and chemical traits (Edmunds 1974; Caetano and Machado 2013; Vickers and Taylor 2020; Kikuchi et al. 2023; Ximenes and Willemart 2024). Nonetheless, it remains unclear how interspecific variation in secretion propensity interacts with other defensive traits.

Variation in the use of chemical secretions may also be associated with other morphological and behavioral traits. For example, body size can influence the balance between chemical and mechanical defenses, including escape‐based strategies such as autotomy: larger individuals may rely more on intimidation or escape, whereas smaller ones may depend proportionally more on chemical or other specialized defenses (Edmunds 1974; Whitman and Vincent 2008; Boevé 2021). One specialized mechanical defense is autotomy, the release of appendages to escape predators (Emberts et al. 2019). Species can also differ substantially in autotomy behavior. For example, the time required for hemipteran leaf‐footed bugs and their allies to release a leg varies by up to an order of magnitude across species (Emberts et al. 2020). Autotomy leaves a visible record of past predation attempts on individuals (Maginnis 2006; Emberts et al. 2020; Powell et al. 2023) and can incur locomotor and energetic costs (Emberts et al. 2019; Escalante et al. 2020, 2021). Because of these costs, individuals may balance the use of mechanical and chemical strategies depending on their ecological context. Consequently, examining correlations between chemical defenses and morphological traits, such as body size and prior injury, can reveal whether sampled species adopt integrated defensive phenotypes or more flexible, context‐dependent strategies.

Arachnids of the order Opiliones are particularly suited for examining how chemical defenses interact with other traits because closely related species often differ in chemical secretion behavior, autotomy, body size, and social aggregation. This variation among sympatric species provides an opportunity to examine whether defensive, morphological, and social traits may be associated with alternative defensive strategies or coordinated defensive phenotypes. Opiliones possess diagnostic paired prosomal scent glands that release chemical secretions (Holmberg 1986; Hara and Gnaspini 2003; Raspotnig 2012). The chemical composition of these secretions varies substantially across lineages, encompassing benzoquinones, naphthoquinones, phenols, ketones, and a diverse array of volatile compounds (Hara et al. 2005; Raspotnig 2012; Raspotnig et al. 2015). Previous work has characterized the chemical composition of Opiliones scent‐gland secretions and tested their antipredator effects against a range of invertebrate and vertebrate predators (Machado et al. 2005; Hara et al. 2005; Escalante et al. 2022; Ximenes and Willemart 2024). By comparison, far less is known about the tendency to release chemical secretions when disturbed, or how this behavioral response varies among closely related and sympatric species, although some Opiliones species are known to release secretions only under strong disturbance (Machado and Pomini 2008). A remaining question is whether these species may differ similarly in secretion propensity and whether such variation is associated with morphological traits (e.g., body size), indicators of prior predation or mechanical defense (e.g., leg loss), or patterns of social behavior (e.g., aggregation tendency).

Aggregation is one of the most commonly observed social behaviors in Opiliones. Species often form conspicuous aggregations of up to hundreds of individuals, and in some systems these aggregations include multiple species sharing the same roost (Donaldson and Grether 2007; Proud et al. 2012; Escalante et al. 2022). Mixed‐species aggregations (MSA) are especially common in Neotropical forests and have been proposed to confer benefits such as predator dilution, enhanced aposematic visibility, microclimate buffering, and information transfer (Donaldson and Grether 2007; Grether et al. 2014; Escalante et al. 2022). Species of *Prionostemma* vary in how often they roost alone or in aggregations, either with conspecifics or heterospecifics (Escalante et al. 2022). Our previous work shows that some sympatric species of *Prionostemma* are found almost exclusively in aggregations, whereas others predominantly roost solitarily (Escalante et al. 2022). This variation in social behavior allows comparisons of chemical secretion behavior among these closely related and sympatric species that differ in their degree of sociality. Exploring whether interspecific differences in secretion propensity correlate with morphological, behavioral, and social traits can help assess whether these traits evolve in a coordinated manner or become decoupled across species.

In this study, we investigated the variation in secretion propensity across 11 *Prionostemma* species from Neotropical forests in Costa Rica. We had two aims: Aim 1: to determine whether species differ in their propensity to secrete when subjected to a standardized assay intended to mimic an acute predatory attack; and Aim 2: to examine potential morphological and behavioral correlates of this variation, with the goal of identifying factors that influence secretion propensity and potential defensive trade‐offs in these arachnids. Under the second aim, we tested three hypotheses: (1) A possible trade‐off between defensive strategies affects secretion propensity and leg loss, which is used here as an indicator of prior injury and potential reliance on mechanical escape. We expected that species with higher leg‐loss frequencies would rely less on releasing chemical secretions. (2) Morphological traits affect secretion propensity, if traits like body size mediate investment and use of chemical versus alternative defense. We expected that smaller species would be more likely to release chemical secretions, as they may rely more on this defense than on locomotion to escape (and vice versa for larger species). (3) Social context affects secretion propensity, as aggregation itself may provide collective safety. We expected that species previously found to roost more frequently in aggregations (Escalante et al. 2022) would rely less on releasing chemical secretions. By combining secretion propensity data and aggregation data, we explored whether chemical secretions contribute not only to predator deterrence but also to the dynamics of social living within this species assemblage.

## 2 Methods

### 2.1 Study System and Field Sampling

**FIGURE 1:**
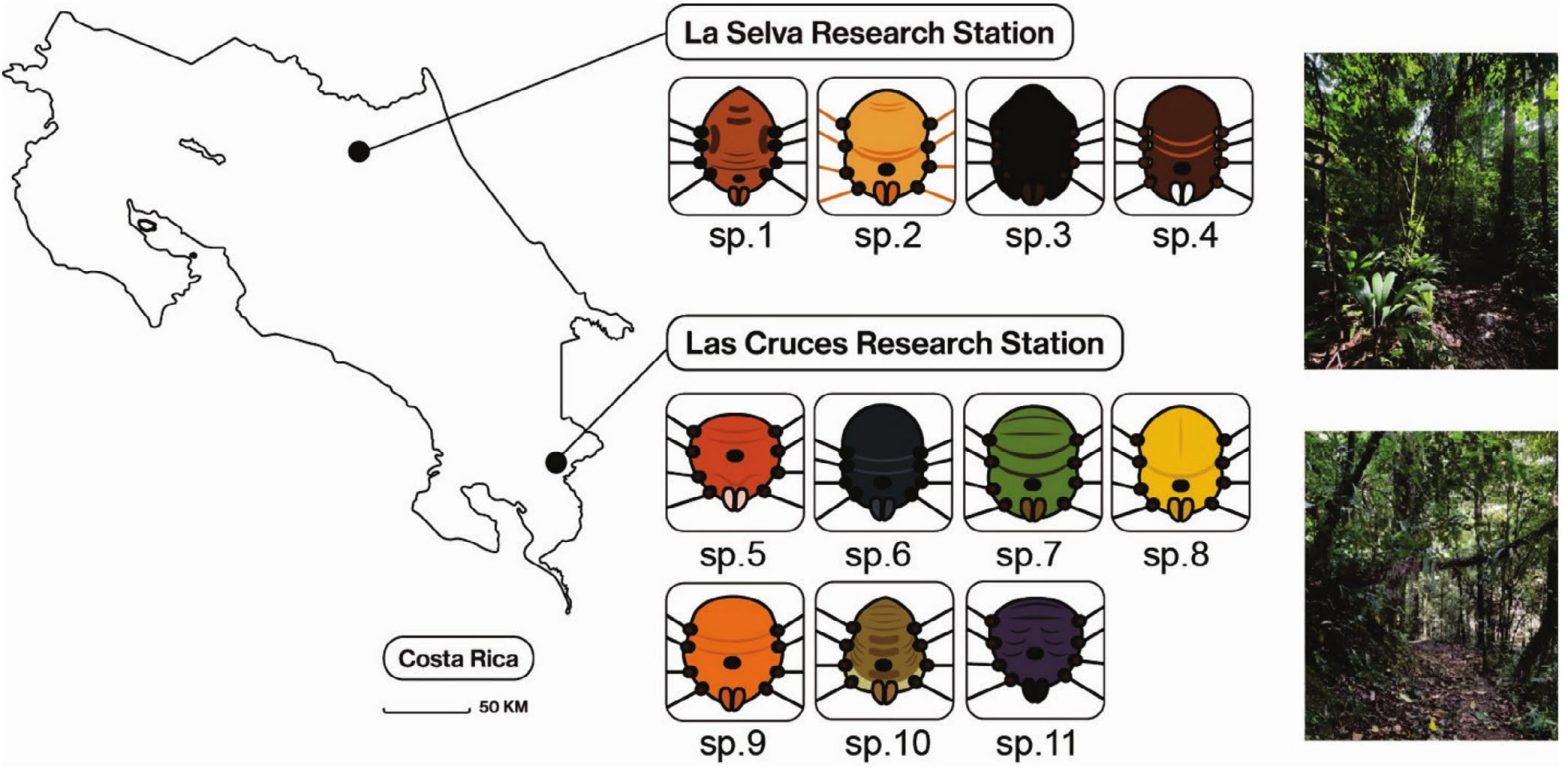
The two sampling sites and 11 study species of *Prionostemma* Opiliones in Costa Rica. Species codes (sp.1–sp.11) are the *Prionostemma* species sampled at each site. Species' illustrations are simplified schematics showing approximate dorsal coloration and are not to scale and are not intended as diagnostic morphological figures.

We conducted fieldwork in 2025 at two Neotropical rainforest sites in Costa Rica. Specifically, in May, we visited La Selva Research Station (Sarapiquí, Heredia province; 10.4315° N, 84.0031° W; 50 m elevation), a lowland rainforest, and in June, we visited Las Cruces Research Station (Coto Brus, Puntarenas province; 8.7844° N, 82.9594° W; 1200 m elevation), a premontane forest. Our study focused on 11 *Prionostemma* species (Opiliones: Eupnoi: Sclerosomatidae), here referred to as sp.1–sp.11. Species sp.1–sp.4 occur at La Selva and species sp.5–sp.11 at Las Cruces (Figure 1). These taxa have been delimited and characterized in previous taxonomic and ecological work (Wade et al. 2011; Proud et al. 2012; Grether et al. 2014; Escalante et al. 2019; Escalante et al. 2020; Villaseñor‐Amador and Escalante 2025, 2026; Domínguez et al. 2016; Escalante and Elias 2021; Escalante and Elias 2022; Escalante et al. 2022). Although the 11 species are currently undescribed, we are confident they represent distinct taxonomic units, as they differ in traits commonly used in the taxonomy of Neotropical Sclerosomatidae (body size and color, relative leg length, and the anatomy of male and female genitalia) (Tourinho‐Davis and Kury 2003; Tourinho et al. 2015). These species are currently being described by combining behavioral, morphological, chemical, and genomic data (Derkarabetian et al. 2022, 2023; Giribet et al. 2022) and can be reliably identified in the field because interspecific variation does not overlap with intraspecific variation (Figure 1). The phylogenetic relationships among these species are currently unknown, which limits inference about the potential evolutionary trajectory of the traits examined here. However, this does not hinder our analyses of behavioral and morphological correlates of chemical secretion propensity.

These Opiliones species roost during the day, typically motionless on substrates such as buttresses, palm fronds, and tree trunks, and forage at night in the understory (Donaldson and Grether 2007; Grether et al. 2014; Escalante et al. 2022). Individuals were collected during the daytime (09:00 to 15:00 h.) from their roosting substrates by grasping several legs simultaneously to minimize injury, placed in plastic containers (30 cm diameter), and transported for less than 1 h to a laboratory at the field station. A total of 483 individuals were sampled across the 11 species (sp.1 = 70, sp.2 = 34, sp.3 = 20, sp.4 = 14, sp.5 = 74, sp.6 = 61, sp.7 = 32, sp.8 = 86, sp.9 = 33, sp.10 = 33, sp.11 = 26). Individuals were housed for less than 24 h in temporary mesocosms (60 × 40 × 30 cm; maximum 15 individuals per container) containing leaf litter and small branches. Containers were kept humid, and animals were provided ad libitum food (cucumber and carrot). To mimic natural conditions prior to experimentation, individuals were allowed to roost either in groups or alone within the mesocosms. All procedures followed established protocols and guidelines for the care and welfare of invertebrates in research (Escalante and O'Brien 2024; ASAB 2025) and were conducted in accordance with Costa Rican governmental regulations for animal research (permits number: R‐032‐2025‐ and R‐025‐2026‐OT‐CONAGEBIO).

### 2.2 Chemical Secretion Assay

Chemical secretion was quantified using a standardized assay intended to mimic an acute predatory attack (Raspotnig et al. 2015, 2022). One individual was tested at a time, randomly selected from the temporary mesocosm. The trial was conducted immediately after grabbing the animal from the temporary mesocosm, on a laboratory bench approximately 10 m away, which was disinfected with 70% ethanol. Each individual was restrained by gently securing as many legs as possible with both gloved hands, keeping their dorsum upward. The individual was then subjected to gentle dorsoventral pressure with the experimenter's thumb for approximately 3 s, or until secretion occurred, and all trials were performed by the same experimenter using the same procedure.

Nitrile gloves were always used while handling Opiliones. The experimenter conducting the assays changed their gloves regularly, specifically every five trials in which no animal released chemical secretions, or immediately after an individual secreted. This way, we certified that no animal was tested with remains of chemical secretions in the experimenter's hand. Changing gloves after every trial could have been ideal. However, we consider that any potential effect is negligible, as there was no indication that the Opiliones' responses were influenced by the immediately preceding trials in which the animal did not release chemical secretions.

Secretion release was recorded as a binary response (yes/no). A trial was scored as “yes” when a visible droplet of fluid emerged from one or both ozopores, accompanied by a detectable odor, and as “no” when no visible droplet emerged and no odor was detected (“no” also includes the instances where individuals released odorless fluids from their mouth, which are mostly water). Each individual was tested once, marked on the dorsal surface with a nontoxic Posca marker to prevent resampling, and released at the capture site. We conducted trials from 08:00 to 20:00 h. The time of day in which the trials were conducted did not affect the likelihood of animals secreting (proportion chi‐square comparing trials in morning, afternoon, and evening: *χ* ^2^ = 1.60, df = 2, *p* = 0.46). All assays were conducted within 24 h of collection.

### 2.3 Autotomy Scoring and Morphological Measurements

Each individual was inspected for preexisting autotomy immediately after the secretion assay. Autotomy was scored as a binary variable (yes/no), with “yes” recorded when one or more legs were missing and “no” when all legs were intact. No autotomy was experimentally induced. Leg loss is common in these species (Escalante and O'Brien 2024). Because our objective was to compare species‐level patterns in secretion propensity, leg loss was treated as a categorical variable rather than a continuous measure (e.g., number of legs lost), as previous work has found that both approaches yield similar behavioral patterns (Escalante and O'Brien 2024).

Body size was quantified by measuring the length of the fourth leg (Leg IV) on one side of the body using digital calipers (0.01 mm precision), a standard body size proxy in *Prionostemma* (Escalante et al. 2020). Measurements were taken while gently restraining the animal to ensure the leg was fully extended without applying force.

### 2.4 Aggregation Status Data

Aggregation data were obtained from a previously collected field dataset from Las Cruces Research Station (Escalante et al. 2022). This was a different dataset from the secretion and morphological datasets and included observations of the seven *Prionostemma* species occurring at Las Cruces (sp.5–sp.11). Aggregation data were unavailable for sp.1–sp.4. For each observation, field records documented species identity and social context at the time of encounter. Observations were classified as solitary (one individual), single‐species aggregation (SSA; two or more individuals of the same species), or mixed‐species aggregation (MSA; two or more individuals of different species) (Escalante et al. 2022).

### 2.5 Statistical Analyses

#### 2.5.1 Secretion Differences Among Species

To address Aim 1 (whether species differ in secretion propensity), we tested for interspecific differences in secretion responses among all 11 species using a chi‐square test, computing both the standard Pearson chi‐square and a Monte Carlo version with 10,000 replicates to account for uneven group sizes. To estimate each species' secretion probability and associated uncertainty, we used logistic regression to model individual secretion response as a function of species identity. Because some species had small sample sizes or complete separation (e.g., all individuals secreted), we modeled individual secretion responses using Firth's bias‐reduced logistic regression (Firth 1993; Heinze and Schemper 2002). Sp.4, which showed the lowest secretion probability, was used as the reference. Likelihood‐ratio and Wald tests evaluated species effects on the model‐estimated secretion probability. Predicted probabilities and 95% confidence intervals were extracted for each species, and pairwise contrasts were computed with Holm correction for multiple testing.

#### 2.5.2 Clustering of Predicted Secretion Probabilities

As an exploratory analysis, we examined whether the 11 species formed broader “secretion phenotypes” based on their likelihood of releasing chemical defenses. We applied hierarchical agglomerative clustering to the predicted probabilities from the Firth model, using Euclidean distances and Ward.D2 linkage (Murtagh and Legendre 2014). Clustering was descriptive and used solely for visualization, as a single value (probability of secretion) was available per species. Silhouette widths were computed for *k* = 2–4 clusters to assess partition coherence.

#### 2.5.3 Autotomy, Body Size, and Their Relationships to Secretion

To address Aim 2 (morphological and behavioral correlates of secretion), and specifically Hypothesis 1 (autotomy‐related defensive trade‐offs) and Hypothesis 2 (body size effects on secretion propensity), we tested species‐level associations between secretion probability, autotomy frequency, and body size. Species‐level differences in autotomy frequency were tested using chi‐square tests on counts of individuals with and without autotomy per species, computing both standard and Monte Carlo versions to reduce reliance on asymptotic *p*‐values. Leg IV length, used as a proxy for body size, was compared among species using a one‐way ANOVA, with a Kruskal–Wallis test as a robustness check. To examine cross‐species associations, the percentages of individuals with autotomy, the mean Leg IV length, and the predicted secretion probabilities were combined. Spearman correlations were computed for all 11 species and interpreted descriptively due to the limited sample size.

#### 2.5.4 Aggregation Analyses

To address Aim 2 (morphological and behavioral correlates of secretion), and specifically Hypothesis 3 (social context effects on secretion propensity), we used species‐level aggregation data for descriptive comparisons with secretion probability. For species with aggregation data (sp.5–sp.11), we calculated the proportion of individuals found in the three aggregation statuses (solitary, SSA, and MSA) from the dataset in Escalante et al. (2022). These species‐level summaries were merged with predicted secretion probabilities to generate descriptive scatterplots. The resulting merged dataset contained one row per species and combined social (aggregation), defensive (predicted secretion probability, autotomy frequency), and morphological (mean Leg IV length) traits. Given the small number of species and nonindependence among group observations, no inferential models were fitted. For visualization, we constructed a species‐level heatmap integrating all traits. For each species, we compiled the mean predicted secretion probability, the mean autotomy frequency, the mean Leg IV length, and the proportions of solitary, SSA, and MSA observations. All numeric variables were *Z*‐scored to facilitate cross‐trait comparison. This visualization allowed an integrated descriptive comparison of all traits across the 11 species. All analyses were conducted in R version 4.4.3 (R Core Team 2024). The Firth logistic regression was performed using the *logistf* package (Heinze et al. 2025), pairwise contrasts using *emmeans* (Lenth and Piaskowski 2025), clustering using *cluster* (Maechler et al. 2026), and visualizations using *ggplot2* (Wickham 2016).

## 3 Results

### 3.1 Secretion Differences Among Species

**FIGURE 2:**
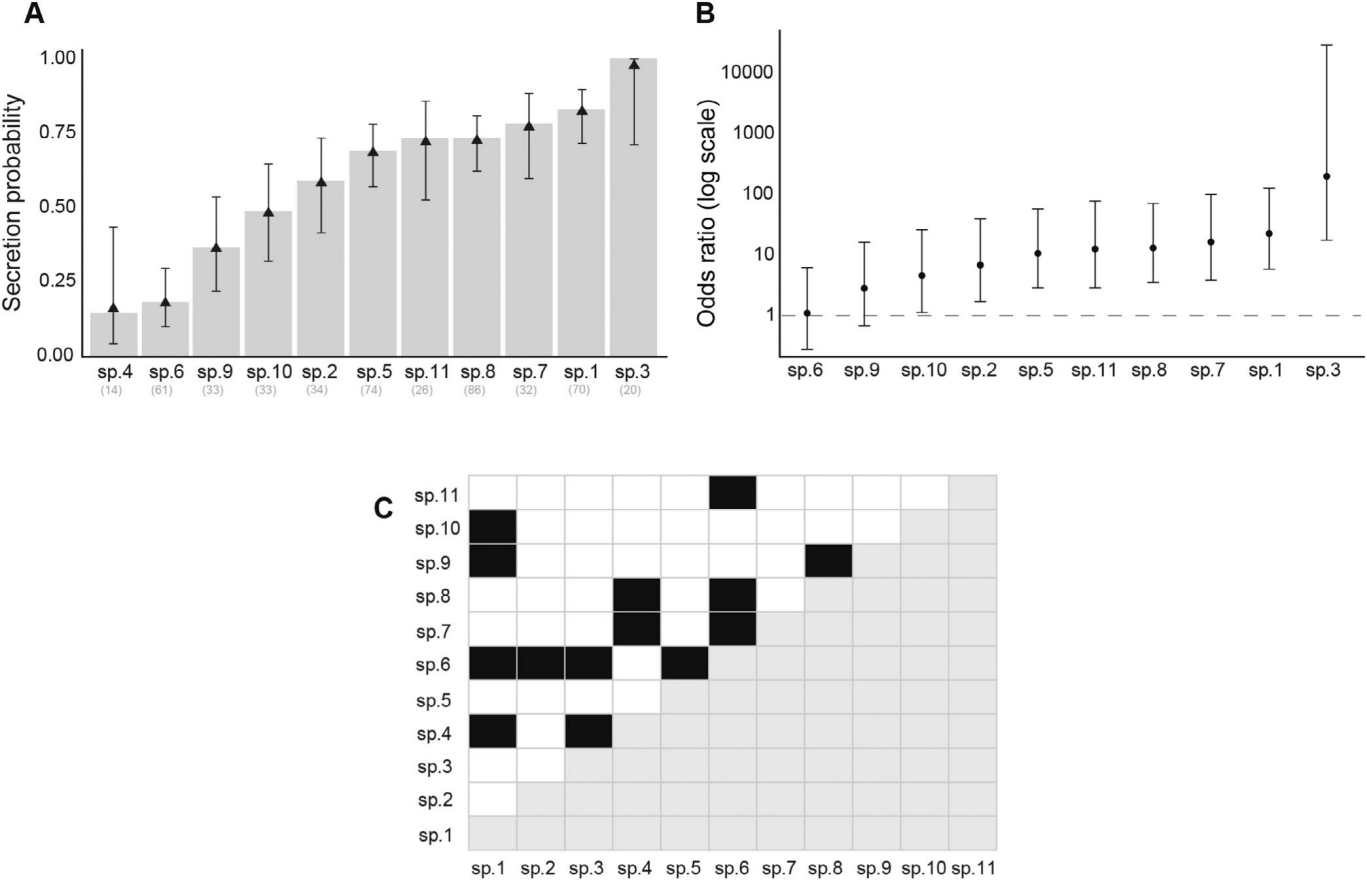
Species in *Prionostemma* differ in their probability of releasing chemical secretions. (A) Bars represent proportions of individuals releasing chemical secretions in each species (sample sizes shown below each species label), and model‐predicted secretion probabilities from a Firth logistic regression (dark triangles with 95% CIs). Species identity was associated with variation in secretion probability. The species with the lowest secretion probability (sp.4) was used as the reference category. (B) Odds ratios (log scale) for each species relative to sp.4. Points show estimated odds ratios and vertical lines show 95% confidence intervals. The dashed line marks an odds ratio of 1, indicating values equal to those of the reference species (sp. 4), with values deviating from this reflecting species with a greater difference. Species are ordered from lowest to highest secreting probability. (C) Holm‐corrected pairwise contrasts from the Firth model. Black cells = significant species‐pair differences at the *p* < 0.05 level.

For Aim 1 (morphological and behavioral correlates of secretion), the proportions of individuals that released chemical secretions when experimentally stimulated varied among species (*N* = 483 individuals in 11 species; species sample sizes: sp.1 = 70, sp.2 = 34, sp.3 = 20, sp.4 = 14, sp.5 = 74, sp.6 = 61, sp.7 = 32, sp.8 = 86, sp.9 = 33, sp.10 = 33, sp.11 = 26; Figure 2A. *χ* ^2^ = 111.07, df = 10, *p* < 0.0001; Monte Carlo version with 10,000 replicates: *χ* ^2^≈111.07, *p* = 0.0001; Table S1). Secretion was least frequent in sp.4 (14%) and most frequent in sp.3 (100%). Several species had high secretion frequencies (sp.1, sp.7, sp.8), whereas others had low values (sp.6, sp.9; Figure 2A). These between‐species differences were also supported by the Firth logistic regression with species as predictor (Figure 2A,B).

Species varied in their probability of secretion (likelihood‐ratio test: LRT = 114.52, df = 10, *p* < 0.0001; Wald test: *W* = 101.04, df = 10, *p* < 0.0001). Using the lowest‐secreting species (sp.4) as a reference, all remaining taxa showed higher predicted secretion probability (positive estimated log‐odds of secretion), though with varying magnitudes. Odds ratios relative to sp.4 ranged from values near 1 (e.g., sp.6, which also had low secretion) to larger contrasts for species with high secretion frequencies (e.g., sp.3; Figure 2B). Confidence intervals were widest for species with small sample sizes (sp.3 and sp.4), but effect sizes were still moderately large (coefficients and odds ratios are reported in Table S2). Thus, despite small sample sizes, both raw data and model‐based analyses consistently support substantial interspecific differences in secretion behavior.

The model‐predicted secretion probabilities closely matched the observed frequencies while incorporating shrinkage for species with limited data (Figure 2A). Predicted probabilities ranged from 0.17 in sp.4 (95% CI: 0.05–0.44) to 0.98 in sp.3 (95% CI: 0.71–1.00), and the remaining nine species varied in their probabilities along this range (Figure 2A). The ranking of secretion probabilities across species based on model predictions also matched the ordering in the raw data (Table S3).

We also found strong support for pairwise species differences in secretion probability (using pairwise contrasts of model‐derived predictions with multiple‐testing Holm adjustment). Fourteen species pairs (out of 55) differed in secretion probability (Figure 2C, Table S3, Figure S1). The significant contrasts primarily involved combinations of the two lowest‐secreting species (sp. 4, sp. 6) versus all other species. Three significant contrasts also occurred among higher‐secreting species (Figure 2C). Model diagnostics based on Pearson residuals and leverage values did not reveal strong deviations from model assumptions (Figure S1). Taken together, the raw frequencies, individual‐level Firth regression, and pairwise contrasts indicate heterogeneity and substantial variation in secretion probability among *Prionostemma* species.

### 3.2 Clustering of Predicted Secretion Probabilities

**FIGURE 3:**
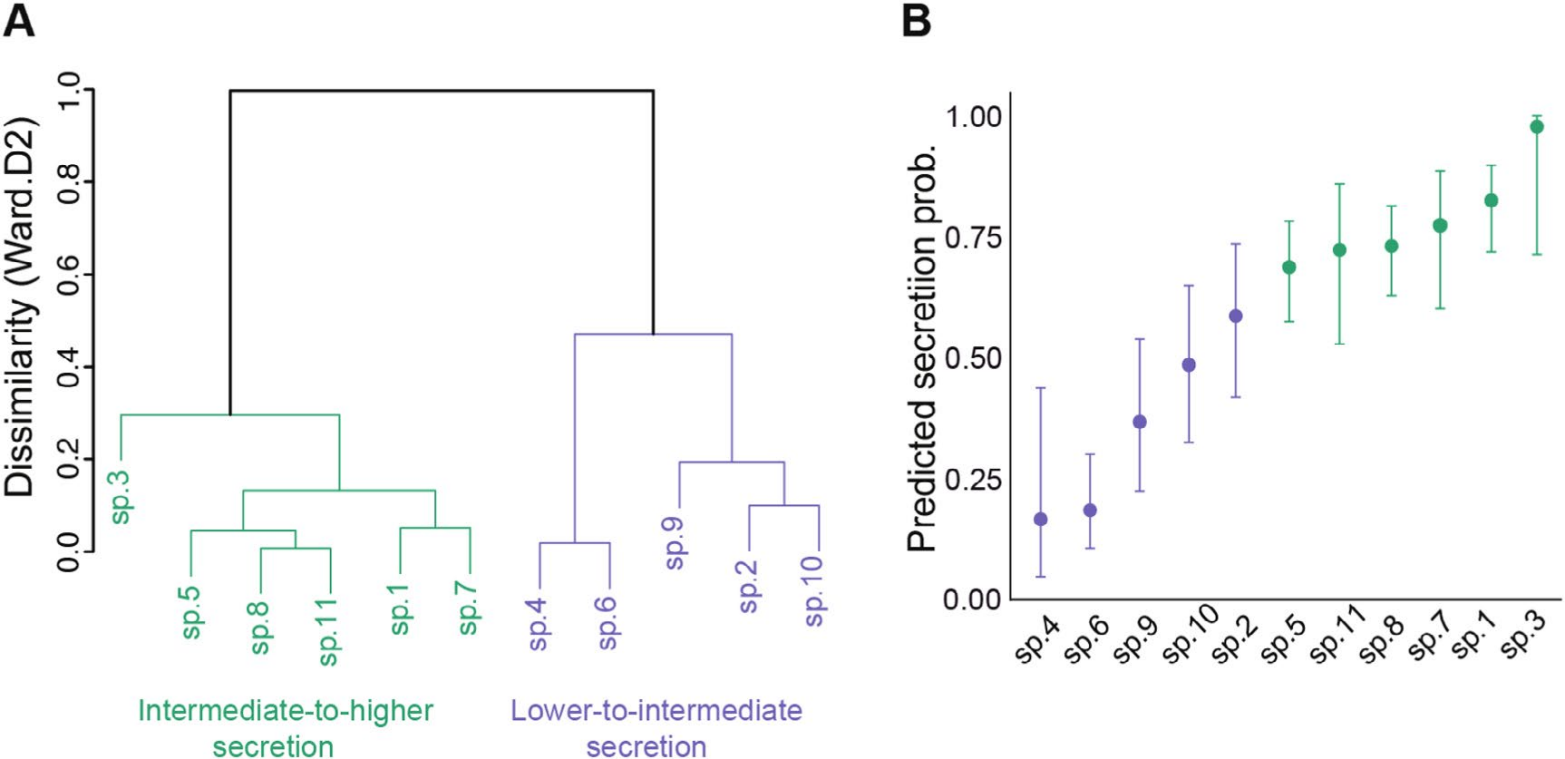
Potential hierarchical clustering of species based on secretion probabilities. (A) Dendrogram constructed using Euclidean distances and Ward.D2 linkage applied to species‐level predicted secretion probabilities. (B) Predicted secretion probabilities with 95% confidence intervals, colored according to the *k* = 2 descriptive partition from the hierarchical clustering. Species assigned by descriptive visualization to the lower‐to‐intermediate cluster are shown in purple, and species in the intermediate‐to‐higher cluster are shown in green. Species are ordered from the lowest to the highest secretion probability.

We found a large continuous gradient from lower to higher secretion, rather than sharply separated groups (Figure 3A shows a tentative two‐cluster arrangement), based on hierarchical clustering (Euclidean distances, Ward.D2 linkage) of model‐predicted secretion probabilities. Species occupied regions of relatively similar secretion levels along this axis (as indicated by low dissimilarity values in Figure 3A). We found limited support for sorting the 11 species into two broad, descriptively distinct secretion phenotypes. We then evaluated the potential optimal number of partitions. For this, the silhouette analyses for partitions of *k* = 2–4 indicated moderate internal coherence across all tested solutions, with average silhouette widths of approximately 0.56 (*k* = 2), 0.59 (*k* = 3), and 0.54 (*k* = 4) (Figures S2–S4, Tables S4 and S5). These similarities suggest that no number of partitions provided stronger support. For descriptive purposes and to facilitate a straightforward low‐versus‐higher secretion contrast, we used the *k* = 2 partition (Figure 3). Under this partition, five species formed the lower‐to‐intermediate secretion cluster (Figure 3, purple group), and the other six formed an intermediate‐to‐higher‐secretion cluster (Figure 3, green group).

### 3.3 Autotomy, Body Size, and Their Relationships to Secretion

**FIGURE 4:**
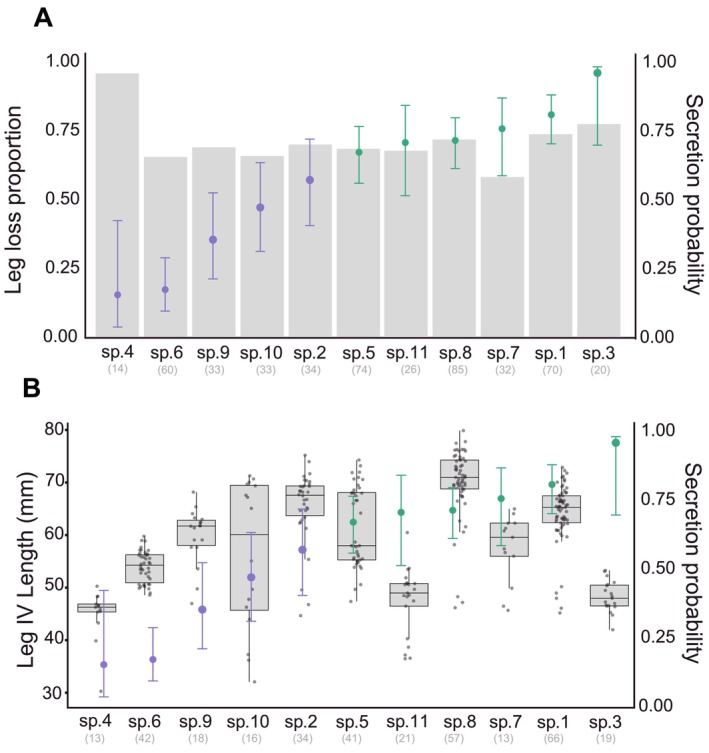
Species‐level leg loss frequencies and body size differences. (A) Leg loss (autotomy) proportion in each species (bars; left *Y*‐axis, sample sizes in parentheses), and model‐predicted secretion probabilities (points with 95% confidence intervals, right *Y*‐axis). (B) Distribution of Leg IV length (mm) for each species (left *Y*‐axis), shown as boxplots with individual measurements, and model‐predicted secretion probabilities (points with 95% confidence intervals, right *Y*‐axis).

Consistent with Hypotheses 1 and 2, interspecific variation in secretion propensity showed no detectable relationship with leg loss levels (Spearman rank correlation *ρ* = 0.14, *p* = 0.68; Figure 4A, Table S6). Levels of leg loss were generally high (56%–93%), but similar across species (*χ* ^2^ = 7.92, df = 10, *p* = 0.64, Figure 4A, Table S7). Additionally, Leg IV length (a proxy for body size) varied among species (range: 30–80 mm; Figure 4B; ANOVA: *F*_10,329_ = 42.31, *p* < 0.0001; Kruskal–Wallis: *χ* ^2^ = 189.84, df = 10, *p* < 0.0001, Table S8). Species‐level mean Leg IV length showed no correlation with secretion probability (Spearman *ρ* = 0.28, *p* = 0.40; Figure 4B). For instance, sp.4 had the highest autotomy percentage, the smallest leg length, and the lowest secretion probability, but this combination was not representative of the 11 species (Figure 4A,B).

### 3.4 Aggregation Behavior and Its Relationship to Secretion

**FIGURE 5:**
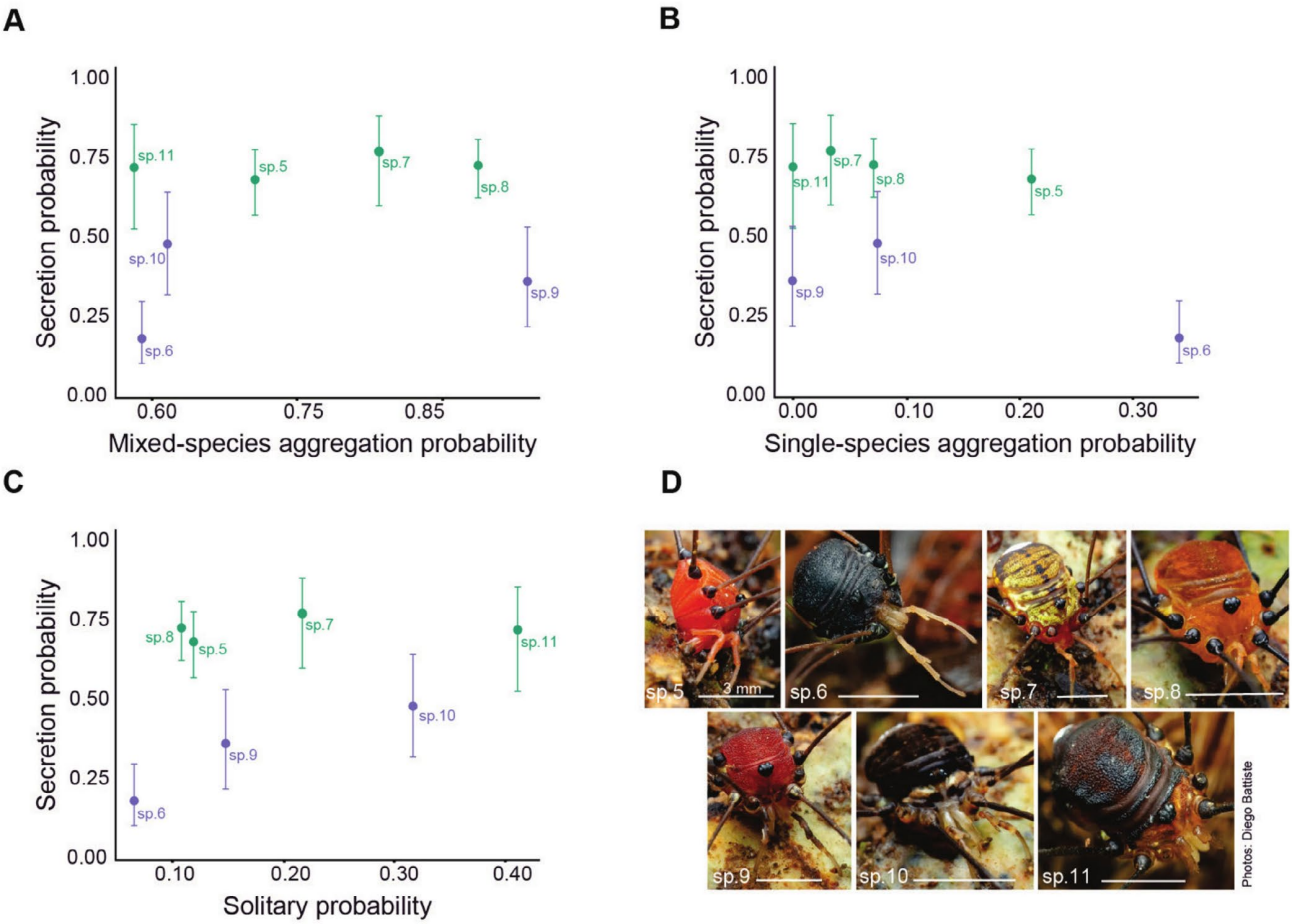
Species differ in their aggregation patterns, and these differences are nonlinear with respect to secretion probability. (A) Predicted secretion probability from the logistic regression (with 95% confidence intervals) plotted against mixed‐species aggregation probability for each species. (B) Predicted secretion probability (with 95% confidence intervals) plotted against single‐species aggregation probability. (C) Predicted secretion probability (with 95% confidence intervals) plotted against solitary probability. In Panels A–C, each point represents a species, aggregation probabilities are derived from Escalante et al. (2022), and species are colored according to the descriptive clustering of secretion propensity (green = intermediate‐to‐higher secreting species; purple = lower‐to‐intermediate secreting species). (D) Photographs of the seven aggregating *Prionostemma* species from Las Cruces Research Station, Costa Rica.

For Hypothesis 3, species‐level predicted secretion probabilities had no straightforward relationship with daytime roosting proportions across three aggregation statuses: MSA, single‐species aggregations (SSA), and solitary (Figure 5). In descriptive analysis, the relationships appeared nonlinear, and species occupied distinct regions of aggregation statuses (Figure 5A–C, Tables S9 and S10). For the MSA status, three intermediate‐to‐higher secreting species (> 67%) had high MSA frequencies, whereas sp.11 had high secretion probability and only moderate MSA (Figure 5A). Among lower‐to‐intermediate secreting species, sp.9 showed high MSA (~85%), whereas sp.10 showed intermediate secretion probability and moderate MSA (~61%). For SSA, species differed markedly: three intermediate‐to‐higher secreting species showed low to intermediate SSA levels (range: < 10%–21%; Figure 5B). Two lower‐to‐intermediate secreting species showed no SSA or very low (~7%), whereas sp.6 exhibited the highest SSA proportion (~34%) and the lowest secretion probability. The solitary status was overall low, with no clear pattern in secretion probability (Figure 5C). For instance, sp. 10 had intermediate secretion with a moderately solitary frequency (~32%), whereas sp. 11 had high secretion with the highest solitary frequency (~41%) (Figure 5C).

### 3.5 Species‐Level Defensive Phenotypes

**FIGURE 6:**
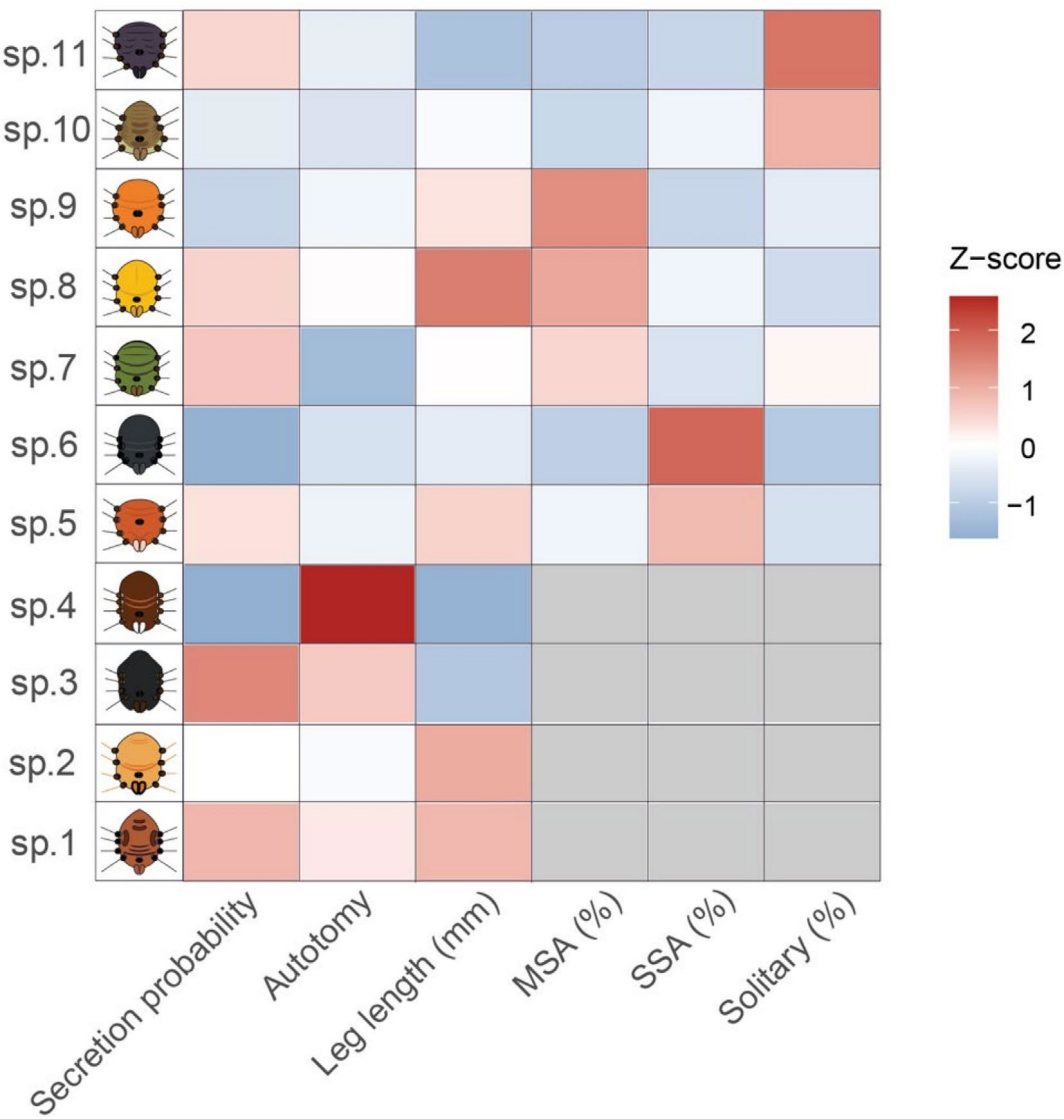
Species of *Prionostemma* differ in their defensive, morphological, and social traits. Heatmap showing *Z*‐scored species‐level values for predicted secretion probability, autotomy frequency, mean Leg IV length, and aggregation statuses (proportion of individuals in mixed‐species aggregations, single‐species aggregations, and solitary). Red colors represent higher relative values within each trait, and blue colors represent lower values; gray cells indicate missing aggregation data (sp.1–sp.4).

We integrated all species‐level trait values into a descriptive overview (Figure 6). There was heterogeneity in species profiles, but secretion probability did not align with any of the phenotypic traits explored (no straightforward color patterns across rows in Figure 6). Specifically, higher secretion probabilities were not consistently associated with lower autotomy frequency, larger body size, or higher proportions of single‐ or mixed‐species aggregations (Figure 6). However, we identified three species‐level combinations that may suggest potential trade‐offs: (1) sp.4 had the lowest secretion probability, above‐average autotomy frequency, and below‐average body size; (2) sp.7 had above‐average secretion probability, below‐average autotomy frequency, average body size, and above‐average MSA (but below‐average SSA); and (3) sp.6 had below‐average secretion probability and autotomy but above‐average SSA. Overall, species‐level profiles show diversity in how *Prionostemma* species combine chemical, morphological, and social traits, but do not support their classification into discrete defensive phenotypes.

## 4 Discussion

### 4.1 Aim 1: Species Differences in Chemical Secretion Propensity

We found substantial interspecific variation in secretion propensity among 11 Neotropical *Prionostemma* species, despite their likely close evolutionary relationships, similar natural histories, and overall morphologies (Wade et al. 2011; Proud et al. 2012; Grether et al. 2014; Escalante et al. 2022). Responses ranged from taxa that rarely secreted during experimental trials to those that secreted in every trial. Descriptive quantitative clustering did not reveal clear discrete biological categories or phenotypes (e.g., “secreting” vs. “non‐secreting”). Instead, secretion propensity varied continuously across species. This variation is consistent with evidence that other traits associated with chemical defenses (e.g., the chemical composition of defensive gland secretions) differ within and between species and lineages in Opiliones (Machado et al. 2005; Hara et al. 2005; Raspotnig 2012; Caetano and Machado 2013; Raspotnig et al. 2017; Solano‐Brenes et al. 2026). More broadly, our findings contribute to our understanding of the variation of chemical defenses in arthropods.

Although we identified interspecific variation in secretion propensity, we found no straightforward relationship with morphological, defensive, or social traits. Consequently, these traits do not appear to explain why the 11 *Prionostemma* species differ in their use of chemical defense. However, work on other Opiliones indicates that secretion production can be both costly and condition‐dependent, potentially interacting with reproductive processes. In Gonyleptidae species, increased food availability enhances benzoquinone production, consistent with a cost of chemical defense that depends on resource state (Nazareth et al. 2016), and egg production trades off with the investment in defensive secretions in Opiliones (Nazareth and Machado 2015). In a Cosmetidae species, juveniles were more likely to release chemical secretions than females, who were more likely to release than males (Albert et al. 2019). Evidence across arthropods suggests that investment in defensive secretions is also constrained by physiological condition and resource availability (Blum 1996; Zvereva and Kozlov 2016; Parvizi et al. 2018; Lindstedt et al. 2020). In addition, Opiliones employ a set of nonchemical defenses, including leg autotomy, crypsis, thanatosis, and rapid escape, which can reduce predation risk potentially independently of secretion (Machado and Pomini 2008; Edmunds 1974; Emberts et al. 2019; Segovia et al. 2019; Ximenes and Willemart 2024; Escalante and O'Brien 2024). Our findings provide insights into factors that appear unrelated to a species' likelihood of using chemical defenses. What explains the variation in multiple antipredator responses of these arachnids remains unknown.

### 4.2 Aim 2: Morphological and Behavioral Correlates of Secretion Propensity

We found that interspecific variation in secretion propensity was not associated with other traits, including leg‐loss frequency, body size, or aggregation tendencies. In particular, Opiliones did not appear to show a simple trade‐off between leg loss and the use of chemical defenses. Frequent leg loss could plausibly predict either reduced reliance on secretion (if escape is favored) or increased reliance on secretion (if mobility is reduced). Body size in arthropods can predict reliance on specific defenses, with smaller‐bodied species relying more on chemical or morphological defenses (Edmunds 1974; Whitman and Vincent 2008; Boevé 2021). However, we did not find overall support for this pattern. In fact, the *Prionostemma* species that secreted the least in our data (sp. 4) was the one with the smallest average leg length. Similarly, secretion propensity showed no simple or linear relationship with interspecific variation in aggregation behavior. Previous work has shown that species vary in their reliance on mixed‐species, SSA, and solitary conditions (Escalante et al. 2022), and our results indicate that such variation does not associate linearly with secretion propensity. Theoretical and empirical work in arthropods indicates that aggregation can interact with chemical defenses: grouping may enhance the effectiveness of warning signals or chemically mediated unpalatability, but it may also arise for reasons unrelated to defense, such as microclimate regulation or mating (Sillén‐Tullberg and Leimar 1988; Codella and Raffa 1995; Wertheim et al. 2005; Cremer et al. 2007; Ruxton et al. 2018; Boulay et al. 2019). However, within the *Prionostemma* species we explored, the coexistence of high‐secreting, highly aggregating species (e.g., sp.7, sp.8) and high‐secreting, largely solitary species (sp.11), as well as low‐secreting species with contrasting aggregation structures (sp.6 vs. sp.9), suggests that secretion propensity and social tendency are not linearly coupled. Whether this decoupling is common across Opiliones or other arthropod groups remains to be explored.

This lack of a simple association between aggregation behavior and secretion propensity suggests that chemical secretions may serve functions beyond direct predator deterrence, a possibility proposed in earlier work for Opiliones scent glands (Holmberg 1986; Schaider and Raspotnig 2009). Chemical secretions can function in social or communicative contexts. Defensive compounds are frequently co‐opted as alarm cues, aggregation signals, or markers involved in group cohesion, particularly in cockroaches, beetles, and aphids (Wertheim et al. 2005; Hoffmann et al. 2006; Boulay et al. 2019). These animals respond to chemical cues produced by conspecifics or heterospecifics, facilitating group formation, coordinated escape, and habitat‐quality assessment. Theoretically, aggregated individuals may reduce their individual investment in costly defenses if they rely on group‐level vigilance, dilution, or social information about risk (Clark and Mangel 1986; Valone 1989; Ward and Webster 2016). Under this perspective, arthropod species embedded in mixed‐species groups may evolve reduced or more flexible reliance on individual‐level chemical defenses, while still achieving effective protection through social mechanisms.

Alternatively, the decoupling between aggregation and chemical defenses may arise because chemical secretion is subject to multiple, potentially conflicting selective pressures. Defensive chemicals can also function as signals or cues in intraspecific and interspecific communication, including aggregation, mate attraction, and roost‐site selection (Wertheim et al. 2005; Donaldson and Grether 2007; Escalante et al. 2022). In many arthropods, aggregation reduces predation risk through dilution, shared vigilance, or confusion effects (Sillén‐Tullberg and Leimar 1988; Uetz et al. 2002; Ruxton et al. 2018; Boulay et al. 2019). Aggregation can also increase the efficacy of chemical defenses. For example, chemically defended caterpillars gain enhanced protection because predators learn to avoid collective signals (Codella and Raffa 1995; Ruxton et al. 2018). If *Prionostemma* scent‐gland secretions have roles beyond antipredator defense, then secretion propensity could be shaped jointly by predator‐mediated selection and social or communicative demands. In that case, correlations between secretion and purely “defensive” traits, such as autotomy, may be weak or context dependent. At present, however, the communicative roles of secretions in *Prionostemma* remain largely speculative, and our data suggest only that secretion propensity varies nonlinearly with morphological and other defensive traits.

### 4.3 Descriptive Trait Combinations Across Species

Although our analyses did not reveal discrete phenotypic clusters, the descriptive patterns in secretion, leg loss, body size, and aggregation indicate recurring trait combinations. Species with similar secretion propensity sometimes differed in their aggregation tendencies, whereas species with similar aggregation tendencies showed different secretion propensities. Together, these patterns may suggest that links between social context and chemical secretion are complex, rather than explained by a single axis of variation.

At a descriptive level, species with high secretion propensity (sp.7, sp.8, sp.11) differed in social strategy, occurring predominantly in MSA (sp.7, sp.8) or showing substantial solitary behavior (sp.11). This pattern may suggest strong chemical defense reliance and flexibility in aggregation strategies. High secretion propensity may either facilitate mixed‐species groups with less chemically defended species or rely on their own chemical protection when roosting alone. Species with intermediate secretion propensity (sp.9, sp.10) also differed in aggregation tendencies despite similar secretion levels. These species are rarely found in SSA or roosting alone, and sp.9 (but not sp.10) roosts almost exclusively in MSA. This pattern may indicate that they join MSA with more chemically defended species. By contrast, the lowest‐secreting species (sp.4 and sp.6) differed markedly in other traits: sp.4 was the smallest species and showed the highest leg loss, whereas sp.6 showed a high aggregation propensity. These combinations may suggest that when chemical defenses are limited, species may rely on alternative strategies, such as mechanical escape, autotomy, or group living.

### 4.4 Next Steps

The ecological, behavioral, and evolutionary roles of chemical secretions in Opiliones remain incompletely understood. Experimental work has emphasized their antipredator function, including evidence that benzoquinone‐rich secretions can deter multiple predator types (Eisner et al. 2004; Machado et al. 2005; Raspotnig 2012; Raspotnig et al. 2017; Escalante et al. 2019; Ximenes and Willemart 2024). However, scent‐gland secretions have also been linked to alarm responses, aggregation dynamics, and reproductive communication (Machado et al. 2002; Raspotnig 2012; Fernandes and Willemart 2014; Escalante et al. 2022). Taken together, our results add a previously underexplored component to the potential multifunctionality of secretions, which bear important implications for our understanding of chemical defenses in arthropods.

Future work combining behavioral assays, chemical profiling, predator trials, and manipulations of social context could help determine whether interspecific differences in secretion propensity reflect variation in defense, communication, or interaction between the two. Future research could also address whether the defensive and morphological traits explored here show phylogenetic signal, as Caetano and Machado (2013) explored in Gonyleptidae, a family of Opiliones. The evolutionary relationships among these *Prionostemma* species remain unresolved, although this aspect is currently under investigation. Importantly, although broader phylogenetic sampling will be needed for macroevolutionary inference, this limitation does not affect our primary conclusions about the comparative patterns among sympatric species. Determining whether more closely related species show similar secretion propensities would be an informative extension of this work, but would be orthogonal to our central finding: that chemical secretion propensity is highly variable across species and does not show a simple or linear association with other behavioral defenses or morphological traits. Together, our findings establish these Opiliones as a promising animal taxon for investigating the ecological and evolutionary drivers of chemical secretion diversity in animals.

## Author Contributions

**Marilia Freire:** conceptualization (lead), data curation (lead), formal analysis (lead), methodology (equal), writing – original draft (lead), writing – review and editing (equal). **Noah Skelly:** data curation (supporting), methodology (supporting), writing – review and editing (equal). **Sejal V. Prachand:** data curation (supporting), methodology (equal), writing – review and editing (equal). **Ignacio Escalante:** conceptualization (equal), funding acquisition (lead), methodology (equal), project administration (lead), supervision (lead), writing – original draft (equal), writing – review and editing (equal).

## Funding

This project was funded by the College of Liberal Arts and Sciences at the University of Illinois—Chicago through start‐up funds to Ignacio Escalante.

## Conflicts of Interest

The authors declare no conflicts of interest.

## Supporting information


**Figure S1:** Diagnostic plots for the Firth logistic regression.
**Figure S2:** Clustering diagnostics *k* = 2.
**Figure S3:** Clustering diagnostics *k* = 3.
**Figure S4:** Clustering diagnostics *k* = 4.
**Table S1:** Model‐predicted secretion probabilities and 95% confidence intervals (CIs) for all species.
**Table S2:** Firth logistic regression coefficients, odds ratios, and 95% CIs (reference: sp.4).
**Table S3:** Pairwise species contrasts from the Firth logistic regression (Holm‐corrected *p*‐values).
**Table S4:** Cluster memberships for *k* = 2, 3, and 4 based on hierarchical clustering.
**Table S5:** Silhouette widths for *k* = 2–4 partitions.
**Table S6:** Integrated autotomy‐secretion data at the species level.
**Table S7:** Species‐level autotomy percentages.
**Table S8:** Species‐level summaries of Leg IV length.
**Table S9:** Species‐level aggregation summaries.
**Table S10:** Integrated aggregation–secretion data.

## Data Availability

The data and the code are publicly and freely available at the DRYAD repository here: https://doi.org/10.5061/dryad.wdbrv1644.
