# Supplementary material for: Chemical Secretion Use Varies Among Species but Lacks a Straightforward Relationship With Other Defenses in Neotropical Opiliones
Source: Ecol Evol. 2026 Jul 19;16(7):e74037. doi: 10.1002/ece3.74037 (PMC13381067; doi:10.1002/ece3.74037)
Supplement: Supplementary file 1 — Figure S1: Diagnostic plots for the Firth logistic regression. Figure S2: Clustering diagnostics k = 2. Figure S3: Clustering diagnostics k = 3. Figure S4: Clustering diagnostics k = 4. Table S1: Model‐predicted secretion probabilities and 95% confidence intervals (CIs) for all species. Table S2: Firth logistic regression coefficients, odds ratios, and 95% CIs (reference: sp.4). Table S3: Pairwise species contrasts from the Firth logistic regression (Holm‐corrected p‐values). Table S4: Cluster memberships for k = 2, 3, and 4 based on hierarchical clustering. Table S5: Silhouette widths for k = 2–4 partitions. Table S6: Integrated autotomy‐secretion data at the species level. Table S7: Species‐level autotomy percentages. Table S8: Species‐level summaries of Leg IV length. Table S9: Species‐level aggregation summaries. Table S10: Integrated aggregation–secretion data. [file ECE3-16-e74037-s001.docx]

**Supplementary Tables**

**Table S1.** Model-predicted secretion probabilities and 95% confidence intervals (CIs) for all species.

| **species** | **pred_prob** | **lower95** | **upper95** |
| --- | --- | --- | --- |
| **sp.1** | 0.823943661971831 | 0.717595138856828 | 0.896044988889614 |
| **sp.2** | 0.585714285714286 | 0.419145820575422 | 0.734745285345938 |
| **sp.3** | 0.976190476190476 | 0.712614068068694 | 0.998527073695555 |
| **sp.4** | 0.166666666666666 | 0.0489221460761057 | 0.437451852095912 |
| **sp.5** | 0.686666666666667 | 0.573626015298633 | 0.781170746460728 |
| **sp.6** | 0.185483870967742 | 0.107164709734202 | 0.301699617860999 |
| **sp.7** | 0.772727272727273 | 0.600994513095458 | 0.884723362353892 |
| **sp.8** | 0.729885057471264 | 0.627331265035914 | 0.812645761735971 |
| **sp.9** | 0.367647058823529 | 0.224528886512322 | 0.538627322918825 |
| **sp.10** | 0.485294117647059 | 0.324886284086908 | 0.64879003741931 |
| **sp.11** | 0.722222222222222 | 0.528309778382048 | 0.857864158984649 |

**Table S2.** Firth logistic regression coefficients, odds ratios, and 95% CIs (reference: sp.4).

| **term** | **estimate** | **se** | **lower95** | **upper95** | **OR** | **OR_low95** | **OR_up95** |
| --- | --- | --- | --- | --- | --- | --- | --- |
| **(Intercept)** | -1.6094379124341 | 0.692820323027555 | -3.24149910474188 | -0.404579678016106 | 0.2 | 0.0391052283029565 | 0.667257214800668 |
| **speciessp.1** | 3.15273602236366 | 0.759667043575027 | 1.79878324064438 | 4.88144065994138 | 23.4000000000001 | 6.04229097570827 | 131.82043557576 |
| **speciessp.2** | 1.95571414915194 | 0.773140352747328 | 0.565161690776443 | 3.70154295451769 | 7.06896551724139 | 1.75973229220057 | 40.509760882454 |
| **speciessp.3** | 5.32300997913841 | 1.59021397547779 | 2.89389026562163 | 10.2945111944235 | 205.000000000001 | 18.0634446889192 | 29569.86934752 |
| **speciessp.5** | 2.39401929895368 | 0.736186570926318 | 1.08824288695962 | 4.08646250681814 | 10.9574468085107 | 2.96905252557208 | 59.5289355924499 |
| **speciessp.6** | 0.129811611521992 | 0.766001633116507 | -1.25910190260308 | 1.8597956220242 | 1.13861386138614 | 0.283908889871529 | 6.42242403526248 |
| **speciessp.7** | 2.83321334405622 | 0.807805062875845 | 1.37953897310192 | 4.6389624080895 | 17 | 3.97306951328668 | 103.436966524344 |
| **speciessp.8** | 2.60347739718263 | 0.733690140985696 | 1.30378609389088 | 4.29250554231612 | 13.5106382978724 | 3.68321530131697 | 73.1495182978625 |
| **speciessp.9** | 1.06711362160874 | 0.778788564314463 | -0.346115408731213 | 2.81734456780746 | 2.90697674418605 | 0.707430838699607 | 16.7323599799269 |
| **speciessp.10** | 1.55059741241117 | 0.773142236428022 | 0.15602064030328 | 3.29477012762302 | 4.71428571428572 | 1.16885032826616 | 26.9712134203092 |
| **speciessp.11** | 2.56494935746154 | 0.815239464584112 | 1.09271909800228 | 4.38116038622591 | 13 | 2.98237242036308 | 79.930730130952 |

**Table S3.** Pairwise species contrasts from the Firth logistic regression (Holm-corrected p-values).

| **contrast** | **odds.ratio** | **SE** | **df** | **null** | **t.ratio** | **p.value** |
| --- | --- | --- | --- | --- | --- | --- |
| **sp.4 / sp.1** | 0.0427350427350426 | 0.0324644035715823 | 472 | 1 | -4.15015505678217 | 0.0019315789301443 |
| **sp.4 / sp.2** | 0.141463414634146 | 0.109371074291085 | 472 | 1 | -2.5295719492617 | 0.375826022674074 |
| **sp.4 / sp.3** | 0.00487804878048778 | 0.00775714134379406 | 472 | 1 | -3.34735454550327 | 0.0370052455912105 |
| **sp.4 / sp.5** | 0.0912621359223299 | 0.0671859589000716 | 472 | 1 | -3.25191927359035 | 0.0503624885107995 |
| **sp.4 / sp.6** | 0.878260869565216 | 0.672749260389279 | 472 | 1 | -0.169466494469272 | 1 |
| **sp.4 / sp.7** | 0.0588235294117646 | 0.0475179448750497 | 472 | 1 | -3.50729832513029 | 0.0218227648821387 |
| **sp.4 / sp.8** | 0.074015748031496 | 0.05430462460839 | 472 | 1 | -3.54846992176414 | 0.0196072594999417 |
| **sp.4 / sp.9** | 0.344 | 0.267903266124175 | 472 | 1 | -1.37022251032676 | 1 |
| **sp.4 / sp.10** | 0.212121212121212 | 0.163999868333216 | 472 | 1 | -2.00557845549229 | 1 |
| **sp.4 / sp.11** | 0.0769230769230768 | 0.0627107280449316 | 472 | 1 | -3.14625268879743 | 0.0685730823297579 |
| **sp.1 / sp.2** | 3.31024390243903 | 1.53432580222953 | 472 | 1 | 2.58252474873812 | 0.343674680930542 |
| **sp.1 / sp.3** | 0.114146341463414 | 0.167210735169834 | 472 | 1 | -1.48153664827531 | 1 |
| **sp.1 / sp.5** | 2.13553398058252 | 0.851713184646248 | 472 | 1 | 1.90236029415372 | 1 |
| **sp.1 / sp.6** | 20.5513043478261 | 9.27892003590016 | 472 | 1 | 6.69528774332768 | 3.37062574841062E-09 |
| **sp.1 / sp.7** | 1.3764705882353 | 0.714762140551417 | 472 | 1 | 0.615328574377285 | 1 |
| **sp.1 / sp.8** | 1.73196850393701 | 0.682745499307758 | 472 | 1 | 1.39334296644036 | 1 |
| **sp.1 / sp.9** | 8.04960000000001 | 3.8064110163775 | 472 | 1 | 4.41056575469195 | 0.000639099498317635 |
| **sp.1 / sp.10** | 4.96363636363637 | 2.30070266599692 | 472 | 1 | 3.45652377487907 | 0.0256561898088016 |
| **sp.1 / sp.11** | 1.8 | 0.955373467047553 | 472 | 1 | 1.10743707389474 | 1 |
| **sp.2 / sp.3** | 0.0344827586206895 | 0.0507556044462135 | 472 | 1 | -2.28770104457973 | 0.65529562569079 |
| **sp.2 / sp.5** | 0.645128891864747 | 0.273489511945055 | 472 | 1 | -1.03390917472192 | 1 |
| **sp.2 / sp.6** | 6.20839580209895 | 2.94165629820121 | 472 | 1 | 3.85358604150843 | 0.00636007747958003 |
| **sp.2 / sp.7** | 0.415821501014199 | 0.224040605663663 | 472 | 1 | -1.62864687534198 | 1 |
| **sp.2 / sp.8** | 0.523214770567472 | 0.219530360923669 | 472 | 1 | -1.54383793555674 | 1 |
| **sp.2 / sp.9** | 2.43172413793103 | 1.20181709649555 | 472 | 1 | 1.79797022201292 | 1 |
| **sp.2 / sp.10** | 1.49947753396029 | 0.727663745333873 | 472 | 1 | 0.834813400103318 | 1 |
| **sp.2 / sp.11** | 0.543766578249336 | 0.299003090486677 | 472 | 1 | -1.10795424900899 | 1 |
| **sp.3 / sp.5** | 18.7087378640777 | 27.1808483648823 | 472 | 1 | 2.01604151961282 | 1 |
| **sp.3 / sp.6** | 180.04347826087 | 264.335404767369 | 472 | 1 | 3.53717845033733 | 0.0199977155305288 |
| **sp.3 / sp.7** | 12.0588235294118 | 17.9726216765225 | 472 | 1 | 1.67054193800784 | 1 |
| **sp.3 / sp.8** | 15.1732283464568 | 22.0251402442572 | 472 | 1 | 1.87349948304657 | 1 |
| **sp.3 / sp.9** | 70.5200000000003 | 104.009042068467 | 472 | 1 | 2.88557422666606 | 0.147093980140834 |
| **sp.3 / sp.10** | 43.4848484848487 | 64.0059363290231 | 472 | 1 | 2.56293085133869 | 0.352711870234117 |
| **sp.3 / sp.11** | 15.7692307692308 | 23.5664064308054 | 472 | 1 | 1.84552933628008 | 1 |
| **sp.5 / sp.6** | 9.62349676225718 | 3.95301068755777 | 472 | 1 | 5.51215189416516 | 3.09660781249845E-06 |
| **sp.5 / sp.7** | 0.644555694618273 | 0.312140486195144 | 472 | 1 | -0.906915428574992 | 1 |
| **sp.5 / sp.8** | 0.811023622047245 | 0.281264921681897 | 472 | 1 | -0.603969611556819 | 1 |
| **sp.5 / sp.9** | 3.76936170212766 | 1.636451333807 | 472 | 1 | 3.05636186007696 | 0.0899618374317955 |
| **sp.5 / sp.10** | 2.32430689877499 | 0.985351483391154 | 472 | 1 | 1.98951474931749 | 1 |
| **sp.5 / sp.11** | 0.842880523731588 | 0.418552819980308 | 472 | 1 | -0.344218484164921 | 1 |
| **sp.6 / sp.7** | 0.0669772859638905 | 0.035397196009945 | 472 | 1 | -5.11527836454477 | 2.37343847854721E-05 |
| **sp.6 / sp.8** | 0.0842753566695252 | 0.0342390067584317 | 472 | 1 | -6.08864234405439 | 1.27507109490521E-07 |
| **sp.6 / sp.9** | 0.391683168316832 | 0.189175318876836 | 472 | 1 | -1.94066236103206 | 1 |
| **sp.6 / sp.10** | 0.241524152415241 | 0.114439490588796 | 472 | 1 | -2.99856356016465 | 0.105663517039132 |
| **sp.6 / sp.11** | 0.0875856816450875 | 0.0472779272169641 | 472 | 1 | -4.51126375314668 | 0.00041530420601825 |
| **sp.7 / sp.8** | 1.25826771653543 | 0.604558346863561 | 472 | 1 | 0.478149589329825 | 1 |
| **sp.7 / sp.9** | 5.848 | 3.19806288034908 | 472 | 1 | 3.2295022215903 | 0.0530617526519795 |
| **sp.7 / sp.10** | 3.60606060606061 | 1.94292034767739 | 472 | 1 | 2.38053546010777 | 0.530503200357482 |
| **sp.7 / sp.11** | 1.30769230769231 | 0.781519203353401 | 472 | 1 | 0.448877967675607 | 1 |
| **sp.8 / sp.9** | 4.64765957446808 | 1.99802258545203 | 472 | 1 | 3.57378132930188 | 0.0182385111381995 |
| **sp.8 / sp.10** | 2.86589297227595 | 1.20248081398463 | 472 | 1 | 2.50934677203516 | 0.385309412004269 |
| **sp.8 / sp.11** | 1.0392798690671 | 0.512225410030553 | 472 | 1 | 0.0781714754728081 | 1 |
| **sp.9 / sp.10** | 0.616631430584919 | 0.304756038118684 | 472 | 1 | -0.978262164804155 | 1 |
| **sp.9 / sp.11** | 0.223613595706619 | 0.124728892040881 | 472 | 1 | -2.68531556074541 | 0.262545899725738 |
| **sp.10 / sp.11** | 0.362637362637363 | 0.199405808948186 | 472 | 1 | -1.84469006233778 | 1 |

**Table S4.** Cluster memberships for k = 2, 3, and 4 based on hierarchical clustering.

| **species** | **k2** | **k3** | **k4** |
| --- | --- | --- | --- |
| **sp.1** | 1 | 1 | 1 |
| **sp.2** | 2 | 2 | 2 |
| **sp.3** | 1 | 1 | 3 |
| **sp.4** | 2 | 3 | 4 |
| **sp.5** | 1 | 1 | 1 |
| **sp.6** | 2 | 3 | 4 |
| **sp.7** | 1 | 1 | 1 |
| **sp.8** | 1 | 1 | 1 |
| **sp.9** | 2 | 2 | 2 |
| **sp.10** | 2 | 2 | 2 |
| **sp.11** | 1 | 1 | 1 |

**Table S5.** Silhouette widths for k = 2–4 partitions.

| **k** | **avg_sil_width** |
| --- | --- |
| **2** | 0.560043915465152 |
| **3** | 0.589153690052266 |
| **4** | 0.538671719268877 |

**Table S6.** Integrated autotomy-secretion data at the species level.

| **species** | **autotomy_percent** | **pred_prob** | **lower95** | **upper95** |
| --- | --- | --- | --- | --- |
| **sp.4** | 92.8571428571429 | 0.166666666666666 | 0.0489221460761057 | 0.437451852095912 |
| **sp.1** | 71.4285714285714 | 0.823943661971831 | 0.717595138856828 | 0.896044988889614 |
| **sp.2** | 67.6470588235294 | 0.585714285714286 | 0.419145820575422 | 0.734745285345938 |
| **sp.3** | 75 | 0.976190476190476 | 0.712614068068694 | 0.998527073695555 |
| **sp.5** | 66.2162162162162 | 0.686666666666667 | 0.573626015298633 | 0.781170746460728 |
| **sp.6** | 63.3333333333333 | 0.185483870967742 | 0.107164709734202 | 0.301699617860999 |
| **sp.7** | 56.25 | 0.772727272727273 | 0.600994513095458 | 0.884723362353892 |
| **sp.8** | 69.4117647058823 | 0.729885057471264 | 0.627331265035914 | 0.812645761735971 |
| **sp.9** | 66.6666666666667 | 0.367647058823529 | 0.224528886512322 | 0.538627322918825 |
| **sp.10** | 63.6363636363636 | 0.485294117647059 | 0.324886284086908 | 0.64879003741931 |
| **sp.11** | 65.3846153846154 | 0.722222222222222 | 0.528309778382048 | 0.857864158984649 |

**Table S7.** Species-level autotomy percentages.

| **species** | **n_total** | **n_autotomy** | **autotomy_percent** |
| --- | --- | --- | --- |
| **sp.4** | 14 | 13 | 92.8571428571429 |
| **sp.1** | 70 | 50 | 71.4285714285714 |
| **sp.2** | 34 | 23 | 67.6470588235294 |
| **sp.3** | 20 | 15 | 75 |
| **sp.5** | 74 | 49 | 66.2162162162162 |
| **sp.6** | 60 | 38 | 63.3333333333333 |
| **sp.7** | 32 | 18 | 56.25 |
| **sp.8** | 85 | 59 | 69.4117647058823 |
| **sp.9** | 33 | 22 | 66.6666666666667 |
| **sp.10** | 33 | 21 | 63.6363636363636 |
| **sp.11** | 26 | 17 | 65.3846153846154 |

**Table S8.** Species-level summaries of Leg IV length.

| **species** | **n_leg** | **mean_leg** | **sd_leg** | **min_leg** | **max_leg** |
| --- | --- | --- | --- | --- | --- |
| **sp.1** | 66 | 63.5484848484848 | 5.78430881248818 | 44.6 | 72.3 |
| **sp.2** | 34 | 64.8823529411765 | 6.99378384551012 | 44.1 | 74.6 |
| **sp.3** | 19 | 47.721052631579 | 2.97070491959371 | 41.4 | 52.7 |
| **sp.4** | 13 | 44.3 | 5.0584912111551 | 29.7 | 49.7 |
| **sp.5** | 41 | 60.9658536585366 | 7.61940318384903 | 46.8 | 73.7 |
| **sp.6** | 42 | 53.3980952380952 | 2.98190578943231 | 48 | 59.2 |
| **sp.7** | 13 | 56.9076923076923 | 6.43252951003226 | 45.1 | 64.4 |
| **sp.8** | 57 | 69.3754385964912 | 6.86963506781199 | 45.6 | 79.3 |
| **sp.9** | 18 | 59.3722222222222 | 5.32549976175178 | 46.4 | 67.6 |
| **sp.10** | 16 | 55.625 | 14.1821719070106 | 31.5 | 70.7 |
| **sp.11** | 21 | 47.052380952381 | 6.32847683472248 | 35.9 | 59.9 |

**Table S9.** Species-level aggregation summaries.

| **species** | **n_total** | **n_solitary** | **n_SSA** | **n_MSA** | **perc_solitary** | **perc_SSA** | **perc_MSA** | **perc_aggregated** | **perc_check** |
| --- | --- | --- | --- | --- | --- | --- | --- | --- | --- |
| **sp.10** | 82 | 26 | 6 | 50 | 31.7073170731707 | 7.31707317073171 | 60.9756097560976 | 68.2926829268293 | 100 |
| **sp.11** | 17 | 7 | 0 | 10 | 41.1764705882353 | 0 | 58.8235294117647 | 58.8235294117647 | 100 |
| **sp.5** | 418 | 50 | 88 | 280 | 11.9617224880383 | 21.0526315789474 | 66.9856459330144 | 88.0382775119617 | 100 |
| **sp.6** | 603 | 39 | 206 | 358 | 6.46766169154229 | 34.1625207296849 | 59.3698175787728 | 93.5323383084577 | 100 |
| **sp.7** | 126 | 27 | 4 | 95 | 21.4285714285714 | 3.17460317460317 | 75.3968253968254 | 78.5714285714286 | 100 |
| **sp.8** | 139 | 15 | 10 | 114 | 10.7913669064748 | 7.19424460431655 | 82.0143884892086 | 89.2086330935252 | 100 |
| **sp.9** | 96 | 14 | 0 | 82 | 14.5833333333333 | 0 | 85.4166666666667 | 85.4166666666667 | 100 |

**Table S10.** Integrated aggregation–secretion data.

| **species** | **n_total** | **n_solitary** | **n_SSA** | **n_MSA** | **perc_solitary** | **perc_SSA** | **perc_MSA** | **perc_aggregated** | **perc_check** | **pred_prob** | **lower95** | **upper95** |
| --- | --- | --- | --- | --- | --- | --- | --- | --- | --- | --- | --- | --- |
| **sp.5** | 418 | 50 | 88 | 280 | 11.9617224880383 | 21.0526315789474 | 66.9856459330144 | 88.0382775119617 | 100 | 0.686666666666667 | 0.573626015298633 | 0.781170746460728 |
| **sp.6** | 603 | 39 | 206 | 358 | 6.46766169154229 | 34.1625207296849 | 59.3698175787728 | 93.5323383084577 | 100 | 0.185483870967742 | 0.107164709734202 | 0.301699617860999 |
| **sp.7** | 126 | 27 | 4 | 95 | 21.4285714285714 | 3.17460317460317 | 75.3968253968254 | 78.5714285714286 | 100 | 0.772727272727273 | 0.600994513095458 | 0.884723362353892 |
| **sp.8** | 139 | 15 | 10 | 114 | 10.7913669064748 | 7.19424460431655 | 82.0143884892086 | 89.2086330935252 | 100 | 0.729885057471264 | 0.627331265035914 | 0.812645761735971 |
| **sp.9** | 96 | 14 | 0 | 82 | 14.5833333333333 | 0 | 85.4166666666667 | 85.4166666666667 | 100 | 0.367647058823529 | 0.224528886512322 | 0.538627322918825 |
| **sp.10** | 82 | 26 | 6 | 50 | 31.7073170731707 | 7.31707317073171 | 60.9756097560976 | 68.2926829268293 | 100 | 0.485294117647059 | 0.324886284086908 | 0.64879003741931 |
| **sp.11** | 17 | 7 | 0 | 10 | 41.1764705882353 | 0 | 58.8235294117647 | 58.8235294117647 | 100 | 0.722222222222222 | 0.528309778382048 | 0.857864158984649 |

**Supplementary Figures**


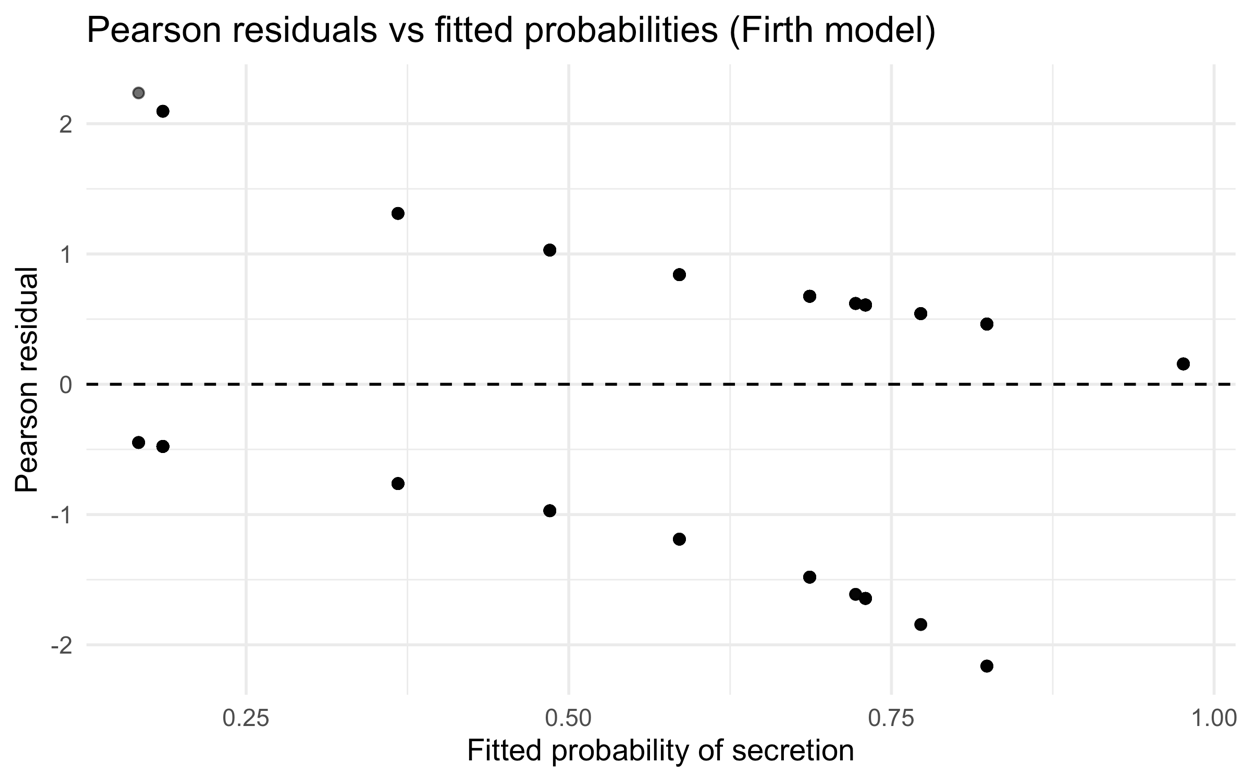


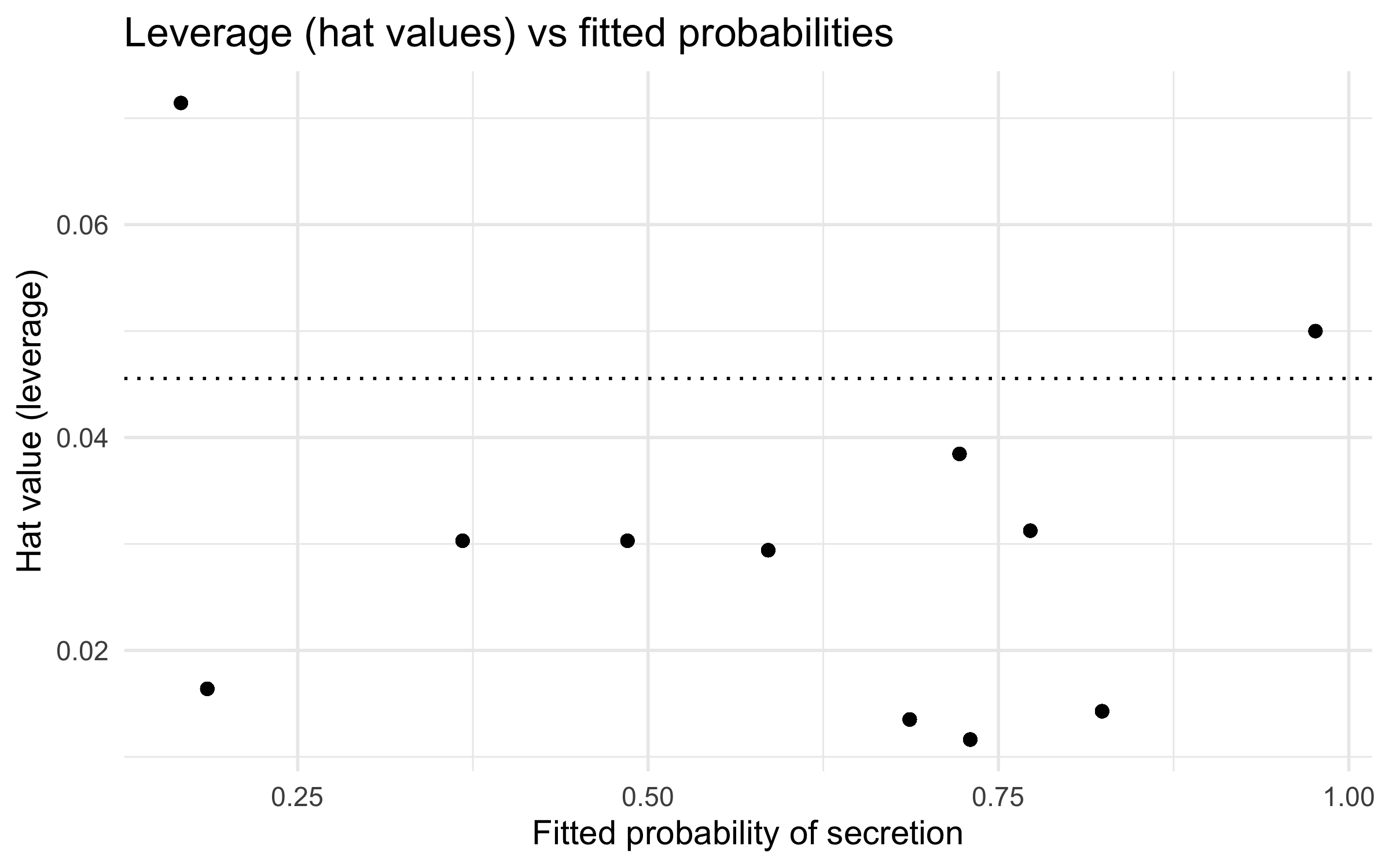


**Figure S1.** **Diagnostic plots for the Firth logistic regression.**

**
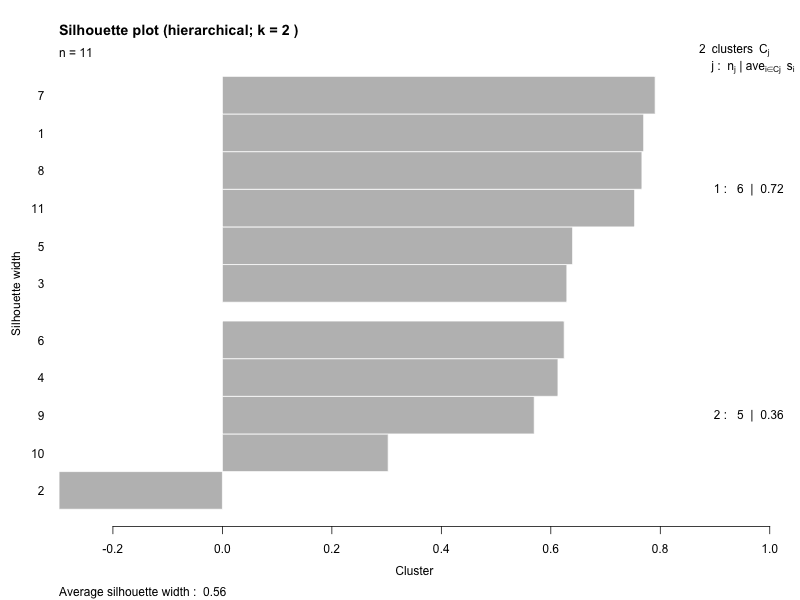
**

**Figure S2.** **Clustering diagnostics k=2**

**
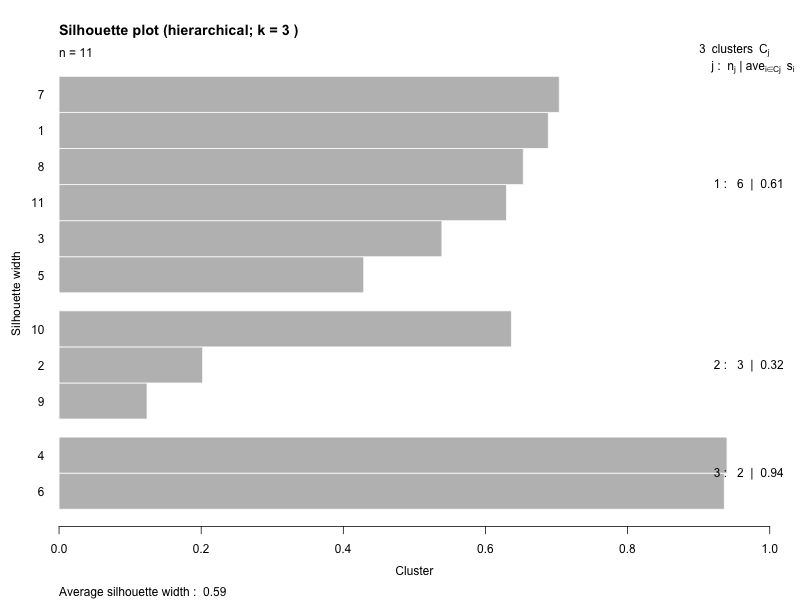
**

**Figure S3.** **Clustering diagnostics k=3.**

**
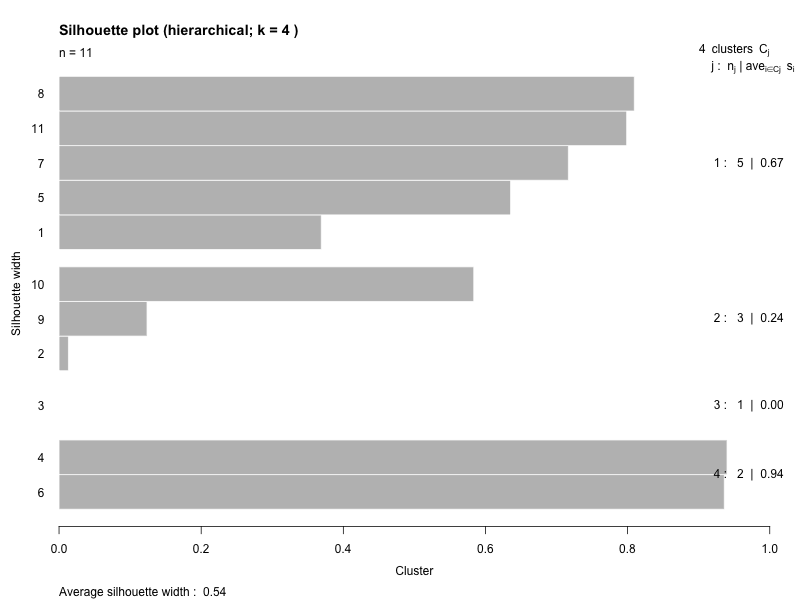
**

**Figure S4.** **Clustering diagnostics k=4.**
